# Molecular characterization of porcine reproductive and respiratory syndrome virus (PRRSv) identified from slaughtered pigs in northern Uganda

**DOI:** 10.1186/s12917-022-03272-x

**Published:** 2022-05-13

**Authors:** Peter Oba, Michel M. Dione, Joseph Erume, Barbara Wieland, Christine Mutisya, Linnet Ochieng, Elizabeth A. J. Cook, Frank N. Mwiine

**Affiliations:** 1 International Livestock Research Institute, P. O. Box 24384, Kampala, Uganda; 2 National Agricultural Research Organization, Abi Zonal Agricultural Research and Development Institute (Abi ZARDI), P. O. Box 219, Arua, Uganda; 3 International Livestock Research Institute, c/o AfricaRice, Rue 18 Cité Mamelles, BP 24265 Ouakam, Dakar, Senegal; 4 College of Veterinary Medicine, Animal Resources and Biosecurity, Makerere University, P. O. Box 7062, Kampala, Uganda; 5 Institute of Virology and Immunology (IVI), Mittelhaeusern, Switzerland; 6 Department of Infectious Diseases and Pathobiology (DIP), Vetsuisse Faculty, University of Bern, Bern, Switzerland; 7 International Livestock Research Institute, P.O. Box 30709, Nairobi, 00100 Kenya

**Keywords:** PRRSv, Species, Porcine, Pigs, Respiratory, Lira, Uganda

## Abstract

**Background:**

A cross sectional study was conducted to detect and characterize species of porcine reproductive and respiratory syndrome virus (PRRSv) identified from slaughtered pigs in Lira district, northern Uganda. The study was conducted from March to September 2019 in three selected slaughter slabs. Pigs brought for slaughter were randomly sampled. At necropsy, lungs were extracted from the thoracic cavity and examined for pneumonic lesions. Seventy-three (73) pigs with gross lung lesions were sampled, from which one hundred and one (101) tissue samples were taken. A real-time reverse transcriptase PCR (RT-qPCR) was used to characterize PRRSv species.

**Results:**

A total of 20 samples tested positive for PRRSv. The respective prevalence of PRRSv type 1 and type 2 were 24.65% (*n* = 18) and 2.73% (*n* = 2) respectively. Of the pigs sampled (*n* = 73), only two pigs, 2.73% (*n* = 2) tested positive to both species. The likelihood of PRRSv detection decreased with pig age, but increased with gross pneumonic pathology.

**Conclusions:**

This study demonstrated dual circulation of both species in northern Uganda. The association between PRRSv and lung pathology suggests that it may be an important cause of lung disease in pigs in Uganda and hence loss of production. This calls for further investigations on potential economic impacts of PRRSv on pig productivity. These findings contribute to discussions about the need of surveillance and possible vaccination strategies against PRRSv in Uganda.

## Background

In Uganda, pig production has increased over the last few years, from approximately 0.7 million in 1990 to 4.2 million pigs in 2018 due to a rising demand for pork [1, 2]. Pig production in Uganda is increasingly becoming an important economic activity for many households, providing a reliable source of livelihoods. However, disease constraints hinder pig production and productivity in the country [3]. Recent multi-pathogen studies reveal occurrence of economically important respiratory pathogens such as porcine reproductive and respiratory syndrome virus (PPRSv), *Mycoplasma hyopneumoniae* (*M. hyo*), *Actinobacillus pleuropneumoniae* (*APP*), *Leptospira* spp. and porcine circovirus (PCV2) type 2 [4–6]. Of the pathogens reported, PRRSv is known to be associated with high economic losses from mortalities, reproductive losses and increased costs of control [7–9]. In general, two genetically distinct species of PRRSv have been described worldwide, with the European species (EU) designated as type 1 (PRRSv-1) and the north American species, designated as type 2 (PRRSv-2) [10]. Furthermore, there is marked genetic diversity in PRRSv-2, leading to further classification into virus lineages [11]. These two species are distinct in their virulence, antigenic characteristics and nucleotide sequences [12, 13]. This has important implications for immunological responses and vaccine selection, as only incomplete protection can be achieved from heterologous field strains [14]. This diversity of the virus also compounds the challenges of disease control, due to differences in transmission rates, strain pathogenicity and its tendency to persist in infected herds.

In the US, PRRSv is reported to cost the swine industry up to $560 million annually, with up to 45% of these losses due to reduced growth and feed efficiency [7]. Overrall, losses due to PRRSv vary widely depending on epidemiological factors, production systems and farm characteristics. In a Dutch study, losses were found to range from €3 to €160 per sow per year﻿ [15]. The economic impacts of PRRSv on swine productivity are justification for epidemiologic studies to generate knowledge to guide interventions.

In Uganda, no vaccines are currently in use for control or prevention of PRRSv. In particular, few studies on PRRSv in Uganda have mainly focused on serologic assays, providing evidence for past exposure of pigs to the virus and possible virus circulation. Recent developments in the pig sector in Uganda show increased imports of breeder pigs from countries such as South Africa, where PRRSv has been reported [16]. This poses a threat to the swine population, if no measures to contain virus spread are established. There is no information on the current epidemiological situation regarding PRRSv and its potential impacts on swine productivity in Uganda, due to lack of surveillance.

Despite the availability of several commercial vaccines in Europe, America and Asia to control PRRSv [17, 18], the apparent lack of information on the identity of current PRRSv strains circulating in Ugandan pigs limits their use as effective tools for control and prevention. The aim of this study was to determine prevalence and to characterize PRRSv species identified from slaughtered pigs in northern Uganda.

## Materials and methods

### Study design

**Fig. 1:**
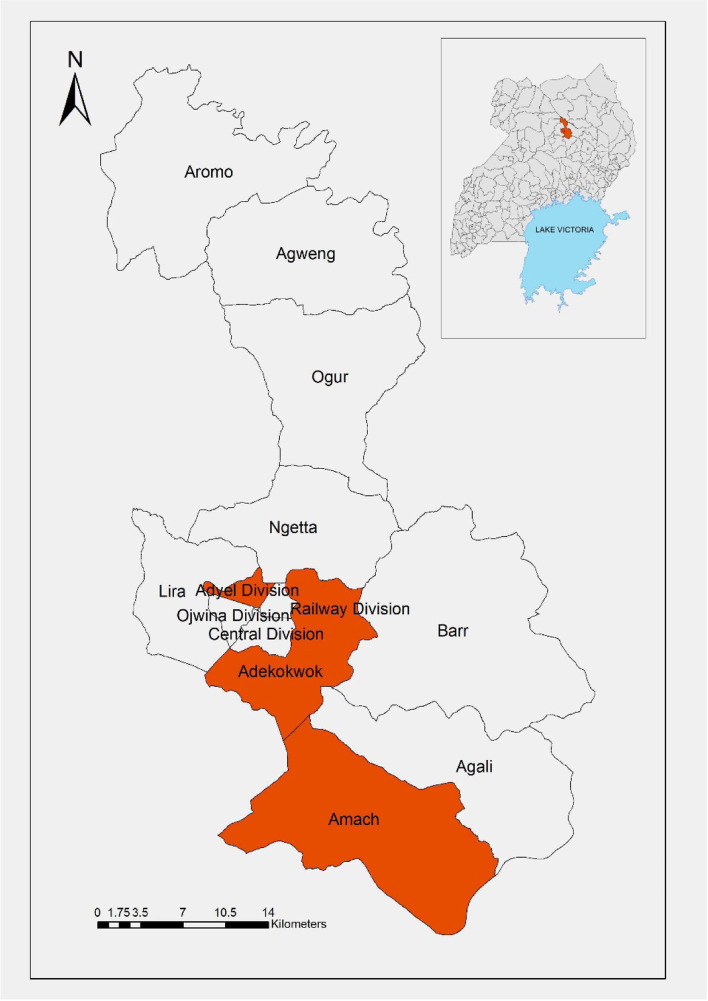
Map of Lira district Uganda showing study sites

A cross-sectional study was conducted from March to September 2019 in three purposely selected slaughter slabs in Lira district, northern Uganda. Slaughter slabs with the highest daily slaughter capacity (≥ 8 pigs) were selected for the study. Pigs slaughtered in Lira district were sourced from within the district (~ 60%), while the rest were sourced from neighboring districts of Apac, Kole, Amolatar and Pader. Figure 1 below shows a map of Lira district in Uganda where the study was conducted.

### Sampling of slaughter slabs and pigs

During this survey, three slaughter slabs: Teso Bar (Adyel division), Adekokwok (Adekokwok subcounty) and Amach market (Amach subcounty) were selected based on high daily slaughter capacity (≥ 8 pigs). In each slab, approx. 40% of pigs brought for slaughter were randomly selected on each day of sampling. At each slab on average, between 8 to 20 pigs were brought for slaughter per day, which represented approx. 8–12 farms. On each day, a list of all pigs brought for slaughter was made and each allocated a number, which were written on a piece of paper and folded. From this list, random sampling was done. Pig biodata was recorded at ante mortem (sex, age), while gross pathology (postmortem) was recorded as described in a related study [19]﻿. Traders were asked about the source(s) of the pigs, which were recorded.

### Sample size determination

The number of pigs sampled represented approximatly 40% of pigs slaughtered in the district *(DVO, pers comm*). The rest (60%) were slaughtered in other smaller slabs distributed throughout the district (~ 30 slabs), and whose daily slaughter capacity varied between 1 and 7 pigs. In a recent study, the seroprevalence of PRRSv in Lira district in pigs was found to be 1.7% [4]. To detect PRRSv from an unknown population size (slaughter-aged pigs), the equation below was used [20].

1 $$n=\ln \left(\alpha \right)/\ln (q)$$

where *n* = is the required sample size, *α* = 0.05, *p* = estimated prevalence of PRRSv (1.7%) and q = 1-p (98.3%). Using this equation, the computed sample size was 175 pigs. Assume 30% of slaughter age pigs show gross pneumonic lesions [19, 21]﻿, a sample size of fifty three (53) pigs was required. During this study, we sampled a total of 73 pigs, from which 101 tissue samples (lungs, lymph nodes, spleen or kidneys) were taken. Only lungs with gross pathologic lesions were sampled, normal lungs or other organs were not sampled. In case a pig had > 1 organ with gross lesions, tissue samples were taken from all grossly affected organs. Out of 73 pigs sampled, 28 pigs had samples (2) taken from 2 organs.

### Examination of lungs and other tissues for gross pneumonic lesions and sample collection

At necropsy, the carcass was placed on a clean table, opened with knives to expose lungs and the pleura. The lungs were carefully extracted from the thoracic cavity and placed on a flat, clean surface. Examination of lungs for gross pneumonic lesion scoring is described in a previous related study ﻿[19]. Lesion samples were taken and cut into ~ 0.5-g pieces, placed in a 2 mL cryovial (Sarstaedt®, Germany) containing RNA*later*® (Thermo Scientific®, USA) tissue stabilization solution. Other observed gross lesions were also recorded. The cryovial was labelled and then placed in an ice box containing ice packs at 4 °C. To prevent cross contamination, a new sterile surgical blade was used for each pig lung, with disinfection of gloved hands and collection tools using 70% ethanol between samplings. Hand gloves were frequently changed to minimize the risk of cross contamination.

### Tissue sample transport and storage

After collection, tissue samples were immediately (within 2 hours) transported to the district (Lira) veterinary laboratory for temporary storage in a fridge at 4 °C. Later, samples were transported (in an icebox at 4 °C) to Makerere College of Veterinary Medicine (CoVAB), Department of Biosecurity, Ecosystems and Veterinary public health laboratory and stored in a − 20 °C fridge. An export permit was secured from the Commisioner Animal Health, Uganda and an import permit from the Directorate of Veterinary Services of the Republic of Kenya to transfer samples to International Livestock Research Institute (ILRI) Kenya for molecular analysis. Upon receipt of an authorization to export samples, tissue samples were shipped by air in October 2019 to ILRI Nairobi, Kenya. The samples were packaged in an ice box containing ice packs at 4 °C, where upon arrival they were placed in a − 80 °C fridge for subsequent RNA extraction and complementary DNA synthesis.

### PRRSv RNA extraction and real-time reverse transcriptase PCR (RT-qPCR)

RNA extraction was done using the AllPrep DNA/RNA Mini Kit (cat. no. 80204) according to the manufacturer’s protocol (Qiagen®, Denmark). A real-time (quantitative) reverse transcriptase PCR was performed in the same laboratory, in March 2020 using the KiCqStart(R) One-Step Probe RT-qPCR ReadyMix™ Low ROX™ (Sigma-Aldrich®). Real-time RT-qPCR and complementary DNA synthesis were performed in a GeneAmp® PCR System 7500 Fast *version 2.3* (Applied Biosystems®). The sequences of primers (Macrogen Europe, cat. no. OG200117–237) for full length cDNA synthesis and the dual-labeled Taq-Man probes are as shown in Table 1 below [22].

**Table 1:** Sequences of primers and dual-labeled probes used in the assay

Genotype	Name	Orientation	Sequence
PRRSv-1	Primer 1	Forward	5′-CGA CCA CCT CAC CCA GAC-3′
	Primer 1	Reverse	5′-CAG TTC CTG CGC CTT GAT-3′
	Probe	Genomic	5′-6-FAM-CCT CTG CTT GCA ATC GAT CCA GAC-BHQ1–3’
PRRSv-2	Primer 2	Forward 1	5′-ATG ATG RGC TGG CAT TCT-3’
	Primer 2	Reverse	5′-ACA CGG TCG CCC TAA TTG-3’
	Probe	Genomic	5′-HEX-TGT GGT GAA TGG CAC TGA TTG ACA-BHQ2–3’

A qPCR master mix was made up of 4 μl molecular biology grade water, 1 μl of 10 μM Forward, 1 μl of 10 μM reverse primers, 1 μl of 10 μM probe and 10 μl of KICqStart Master mix (Sigma Aldrich, UK). The master mix was completely mixed by tapping the tube and a quick short spin. This master mix cocktail was adequate for one reaction. The components of the master mix were adjusted to suit the number of samples. The contents of the master mix tube were mixed thoroughly and dispensed 17 μl to each labeled sample and control tubes. An RNA template of 3 μl was then dispensed to each tube with a master mix. The tubes were placed in a 7500 Fast Thermalcycler and the program which includes a Reverse Transcriptase (RT) at 50 °C for 10 min, pre-heating at 95 °C for 10 min, denaturation at 95 °C for 30 seconds and annealing at 60 °C for 1 minute was started. This was repeated for 45 cycles with the RT and preheating occurring just once.

### Data analysis

Strain identification was determined by plotting amplification curves of fluorescence signal detected versus cycle threshold values (Ct). Cycle threshold values of ≤42 were considered positive and Ct value > 42 were taken as negative. Summary statistics were derived in the R environment for statistical computing, *version 4.0.4* (http://cran.r-project.org/). The relationship between PRRSv positivity age, sex, location and gross pathology was measured using Chi-squared analysis in the *epiDisplay* package in R. An individual pig was the unit of analysis; a pig was considered positive if any of the organs were found positive by RT-qPCR. Odds ratio (OR) values were calculated based on positivity of PRRSv-1.

## Results

Seventy three (73) pigs were sampled, from which 101 tissue samples were taken. Of the pigs sampled (*n* = 73), the prevalence of PRRSv type 1 and type 2 were 24.65% (*n* = 18) and 2.73% (*n* = 2) respectively. Only two pigs, 2.73% (*n* = 2) tested positive to both PRRSv type 1 and type 2, implying that the 2 pigs that had PRRSv-2 were also co-infected with PRRSv-1. There was a significant relationship between PRRSv positivity and the degree of lung pathology, Odds Ratio 3.74 (95% CI 1.14–15.05). In a related study [19], the prevalence of gross pneumonic lesions ranged from 17.3% for pleuritis, 29.9% for catarrhal purulent bronchopneumonia (CPBP), to 74.2% for pleuropneumonia (PLP). Table 2 below shows a summary of results.

**Table 2:** Summary of the Chi^2^ analysis for PRRSv-1 and PRRSv-2 positive samples collected from pigs in Lira District, Uganda

Variable	Category (N = 73)	PRRSv-1 prev. % (n)	PRRSv-2 prev. % (n)	Odds Ratio (95% CI)	Chi^2^ test, df, *p*-value
Pig sex	Males (*n* = 35)	25.71 (*n* = 9)	0 (*n* = 0)	1.12 (0.38–3.32)	0.05, 1, 0.841
	Females (*n* = 38)	23.47 (*n* = 9)	5.26 (*n* = 2)	1	
Pig age	≤12 months (*n* = 48)	29.17 (*n* = 14)	4.17 (*n* = 2)	1	–
	> 12 months (*n* = 25)	16.66 (*n* = 4)	0 (*n* = 0)	0.46 (0.13–1.59)	1.53, 1, 0.216
Gross pathology	0–24% (*n* = 33)	12.12 (*n* = 4)	0 (*n* = 0)	1	–
	25–72% (*n* = 40)	35.00 (*n* = 14)	5.00 (*n* = 2)	3.74 (1.15–15.05)	3.93, 1, 0.023*
Slaughter slab	Teso bar (*n* = 39)	28.20 (*n* = 11)	2.56 (*n* = 1)	1.30 (0.37–5.05)	–
	Adekokwok (*n* = 26)	23.07 (*n* = 6)	3.84 (*n* = 1)	1	
	Amach mrket (*n* = 8)	12.56 (*n* = 1)	0 (*n* = 0)	0.49 (0.01–5.27)	0.936, 2, 0.626
Origin of pig	Lira (*n* = 43)	20.93 (*n* = 9)	4.65 (*n* = 2)	1	
	Neighboring districts (*n* = 30)	30.00 (*n* = 9)	0 (*n* = 0)	1.60 (0.53–4.83)	0.37, 1, 0.37

Gross pathology represents percent estimate of lung surface area grossly affected by pneumonia; neighboring districts are Alebtong, Pader, Dokolo, Kole and Apac

## Discussion

This study revealed circulation of both type 1 and 2 PRRSv genotypes in northern Uganda. However, PRRSv type 1 was found to be the more predominant genotype detected. Given the high animal movements for slaughter, restocking and breeding between regions [23] and the weak surveillance systems, the potential for spread of PRRSv may be substantial. This implies that PRRSv-1 may likely be prevalent elsewhere in Uganda, where its occurrence has not yet been investigated properly. This situation could have adverse implications for swine productivity in the country, herd economic performance and consequently livelihoods, if the virus becomes established in commercial breeding herds. Information about the predominant virus is important for implementing successful interventions for controlling the spread of the virus given its potential economic impacts on swine productivity. However, clinical manifestations and potentially economic impact might be very different between PRRSv-1 and PRRSv-2 infections.

These results showed the likelihood of PRRSv-1 detection decreased with pig age (range 5–50 months). While this was not statistically significant, it suggested a trend that needs further exploration with a larger sample size. This finding is consistent with the observation that the immune system of swine is able to completely eliminate PRRSv infection over prolonged periods of time [18]. Pigs exposed to PRRSv become resistant to reinfection with a homologous strain, although the level of protection was incomplete [24]. This was also corroborated by a study which found age-dependent resistance to infection, shown by reduced viremia and viral load in the blood of adult pigs compared to younger pigs [25]. In contrast, other studies revealed that PRRSv tends to persist in infected herds [26, 27], suggesting increased likelihood of detection in older pigs. However, this finding was specific for larger herds and where there were increased re-introductions of infected gilts [28]. As part of a major longitudinal study (Oba et al. *unpublished*), most farms in the district were generally small in size (1–5 sows) and the replacements were infrequent. The estimated prevalence was obtained from randomly selected clinically healthy growing/adult pigs from households in the region. This implies that there exists age differences between the population in which the expected prevalence is drawn and the population from this study.

The increase in PRRSv detection rates associated with gross pathologic lesions conforms to previous studies. The ability of PRRSv to induce clinical and macroscopic pneumonia, often as a co-infection with other pathogens such as *M. hyo* has been documented [29]. No differences in detection rates between male and female pigs were observed in this study. While PRRSv type 1 was detected in both Lira and the neighboring districts, type 2 was detected only from Lira district. This suggests type 1 may be widespread compared to type 2. Apart from Lira district, pigs were also sourced from Apac, Kole, Amolatar and Pader districts and no localization was observed in any of the districts.

Our results are comparable to other studies which reported simultaneous circulation of both PRRSv type 1 and type 2 species in various regions and show increased circulation of PRRSv type 1. In Europe, both species circulate but there is a predominance of type 1, with marked genetic variation among species [30]. In Asia, studies report the predominance of PRRSv type 1 in China, although the American type 2 has also been documented [31]. In the Republic of Korea, it was found that both type 1 and type 2 species circulated in pig farms during the period between 2013 and 2016. However, type 1 PRRSv was reportedly predominant [32].

The information on PRRSv in African countries is limited but there are official reports submitted to OIE by a few countries in Africa (DR Congo, Benin, Burkina Faso, Egypt, Ivory Coast, Nigeria) that document occurrence of PRRSv, although none of these studies reported its genetic diversity or molecular identity [16]. The current situation regarding the PRRSv species circulating on the continent is largely unknown, as the few studies undertaken were based on serologic assays. In southwest Nigeria, a study reported a high seroprevalence of PRRSv of 53.8%, suggesting widespread exposure of pigs to the virus [33]. However, the species of the virus was not determined.

In South Africa, the PRRSv strain responsible for the 2004 outbreaks was identified by RT-PCR as type 2 [34]. Our results are contrary to expected since a large number of pigs are imported from South Africa and suggest a different source of the virus in Uganda, since PRRSv type 1 has not been reported in South Africa. The lack of reliable data on pig imports into Uganda limits our understanding of the likely sources of PRRSv introduction into the country. Further studies to understand the introduction and maintenance of PRRSv into Uganda are required. Knowledge gaps remain on the potential distribution of PRRSv species in other regions of Uganda especially in high pig dense areas, which justify further studies.

The method used to detect PRRSv in this study utilised primers that were designed to simultaneously detect both PRRSv-1 and PRRSv-2. This approach is reported to have high specificity and sensitivity, at differentiating PRRSv-1 from PRRSv-2 isolates [22, 35]. This method is reportedly efficient and rapid for large scale detection and differentiation of PRRSv species. However, this study was limited by the small sample size used and by the fact that the study was undertaken in only one region, implying that results cannot be extrapolated to other regions of the country. Because we sampled only pigs that presented with gross lung lesions, the true prevalence of PRRSv and the distribution of species in all slaughtered pigs and in the general pig population still remains unknown and possibly is higher to what has been reported here. The future option of sequencing at least a portion of the genome of the PRRSv strains identified could be included with the aim to aid future epidemiology studies.

## Conclusions

This is the first study to document dual circulation of PRRSv type 1 and 2 species in pigs in Uganda. The relation between PRRSv and severe lung pathology suggests it may be an important and increasing cause of lung disease in pigs in Uganda and hence loss of production. This study reveals PRRSv-1 is the predominant genotype in circulation among slaughter-age pigs in Lira district in northern Uganda. However, in view of its reported genetic diversity, further characterization of possible PRRSv-1 subtypes and evaluation of their pathogenicity in pigs is justified, as well as investigate possible circulation of PRRSv in other parts of the country with the aim to establish surveillance. In addition, studies to evaluate efficacy of different control measures, such as vaccination, considering dual circulation of the two species and to quantify their economic effects in Uganda are recommended.

## Data Availability

The datasets used and/or analysed during the current study are available from the corresponding author on reasonable request. Both tissue samples and PCR products for this paper are stored at ILRI laboratory (Lab 5), ILRI campus, Nairobi, Kenya and can be obtained upon request.
